# Supplementary material for: Anti-phage islands force their target phage to directly mediate island excision and spread
Source: Nat Commun. 2018 Jun 14;9:2348. doi: 10.1038/s41467-018-04786-5 (PMC6002521; doi:10.1038/s41467-018-04786-5)
Supplement: Supplementary file 1 — Supplementary Information [file 41467_2018_4786_MOESM1_ESM.pdf]

Anti-phage islands force their target phage to directly mediate island excision and spread

McKitterick and Seed

Supplementary information

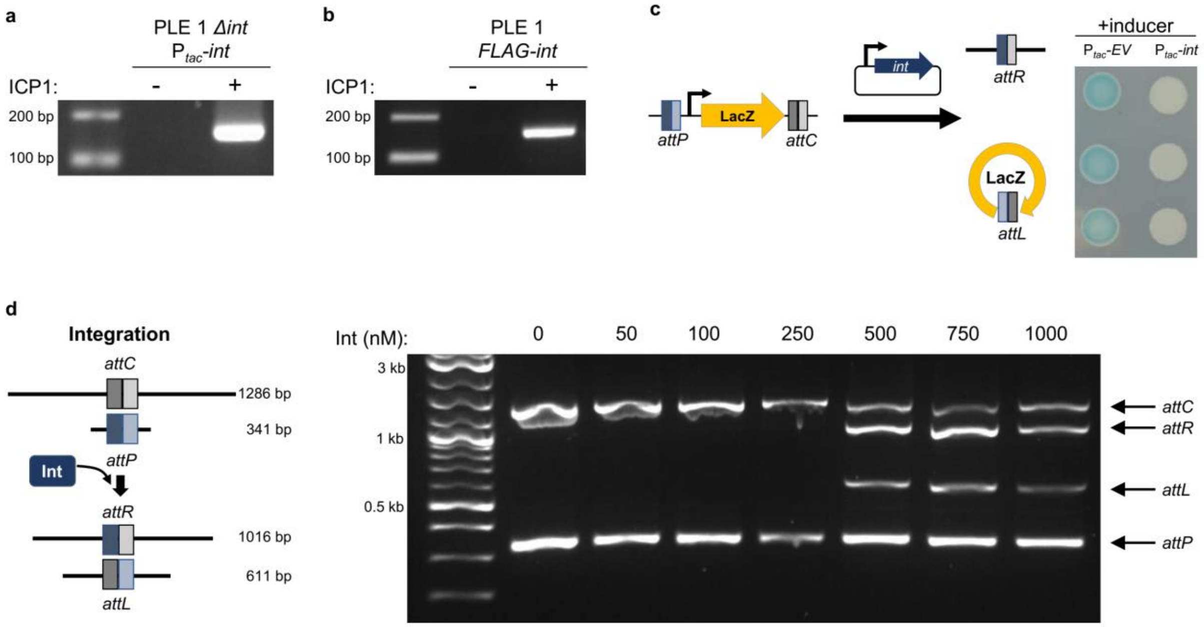

Supplementary Fig. 1 | PLE 1 Int is a functional integrase. a, Ectopic expression of Int is not sufficient to drive PLE circularization in the absence of ICP1 infection. **b,** Endogenously FLAG-tagged PLE Int is still able to drive functional circularization within 5 minutes of ICP1 infection. **c**, Cartoon depicting the *in vivo* integration assay. Constitutively expressed LacZ from *E. coli* was flanked by PLE 1 *attP* and *attC* sites and integrated into the *V. cholerae* chromosome. When ectopically expressed, Int recombines the *att* sites, creating the hybrid *attL* and *attR* sites and excising LacZ from the chromosome. Three independent colonies of each vector type were spotted on indicator plates containing IPTG, theophylline, and X-gal and incubated for 24 hours. **d**, Left, cartoon depicting an *in vitro* integration assay, in which dsDNA containing the PLE 1 *attC* and *attP* sites is incubated with Int to determine if the recombination products *attL* and *attR* are produced. Right, results of the *in vitro* integration assay showing increasing concentrations of Int lead to the production of *attR* and *attL*. Uncropped gels are presented in Supplementary Fig. 5.

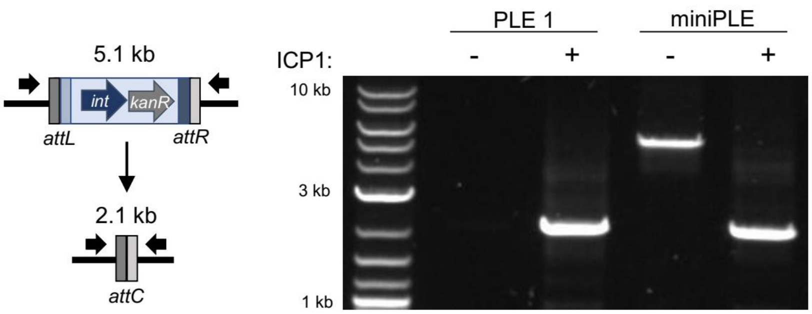

Supplementary Fig. 2 | The miniPLE excises from the chromosome during ICP1 infection. Left, cartoon of primers used to detect PLE and miniPLE excision. Right, PCR to detect excision of PLE 1 or miniPLE during ICP1 infection. PLE 1 is 18kb and cannot be PCR amplified when integrated in the genome. Uncropped gels are presented in Supplementary Fig. 5.

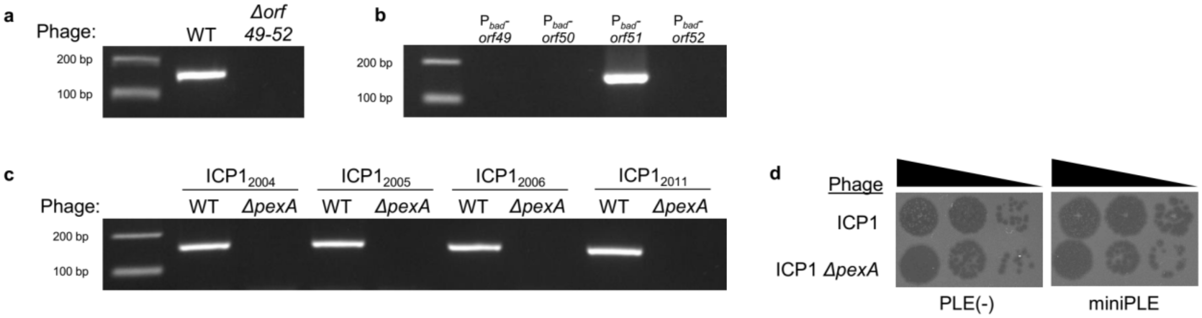

Supplementary Fig. 3 | ICP1-encoded ORF51 (PexA) contributes to PLE circularization. a, miniPLE circularization plaque PCR. Agar stabs from $ICP1_{2004}$ plaques on miniPLE were boiled and used as template to detect circularization. When $ICP1_{2004}$ has a mutation in *orf49-52*, miniPLE circularization is not detected. **b,** Individual ORFs were cloned into an inducible P_{bad} vector and screened for miniPLE circularization. PLE circularization is detected only when *orf51* is induced. **c,** ICP1 *pexA* is necessary for miniPLE circularization in multiple ICP1 isolates. Different IPC1 isolates with and without clean deletions of *pexA* were tested for miniPLE circularization via plaque PCR. **d,** Tenfold dilutions of ICP1 spotted on various *V. cholerae* lawns shows that ICP1 *ΔpexA* does not have any defects in plaque formation (dark spots, zones of killing) relative to wild-type ICP1. Uncropped gels are presented in Supplementary Fig. 5.

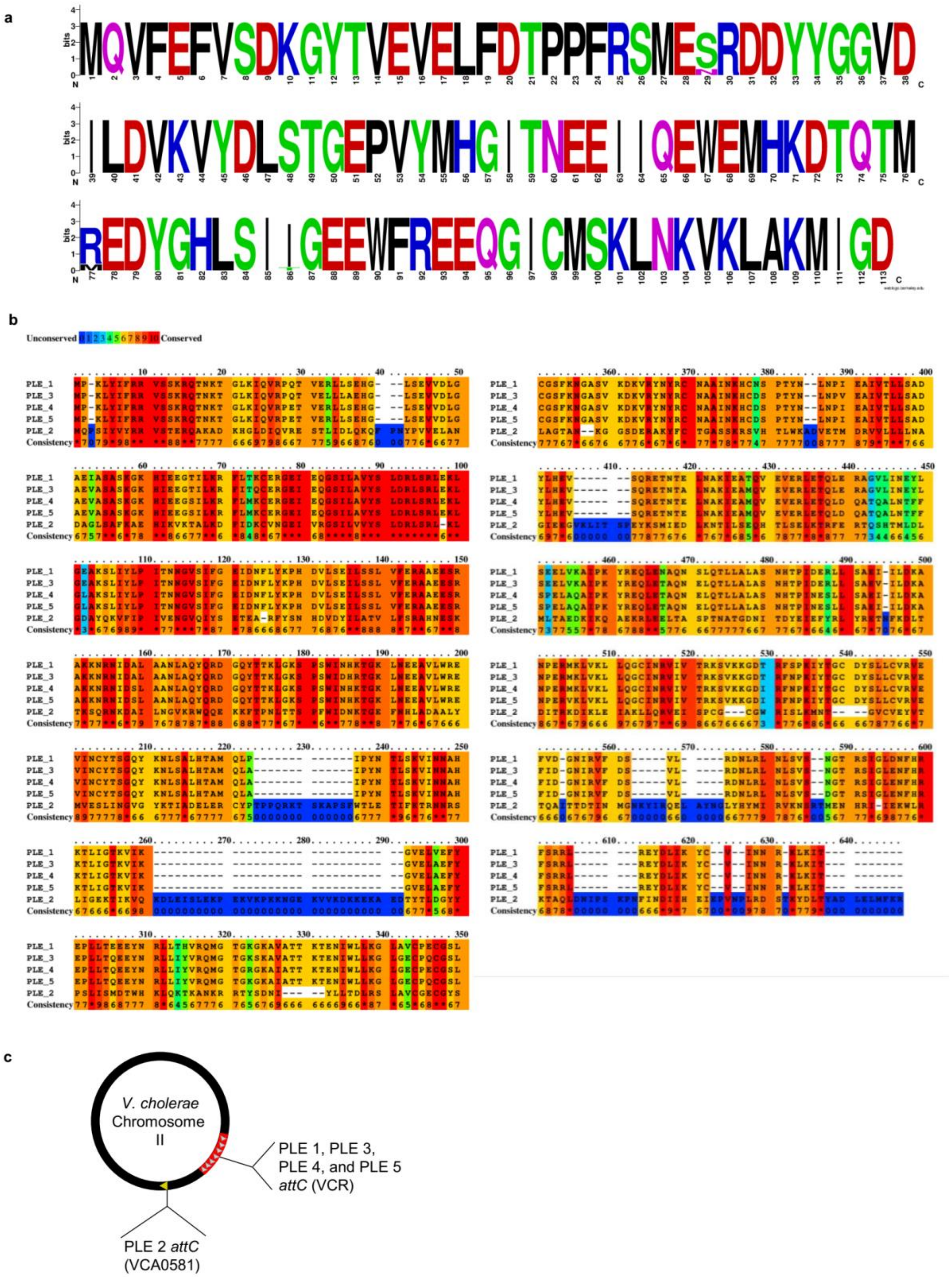
a
b
Unconserved
Conserved
PLE_1
PLE_3
PLE_4
PLE_5
PLE_2
Consistency
c
V. cholerae
Chromosome
II
PLE 1, PLE 3,
PLE 4, and PLE 5
attC (VCR)
PLE 2 attC
(VCA0581)

Supplementary Fig. 4 | The conservation of PLE Int and ICP1 PexA between isolates. a, Sequence logo[1] depicting the amino acid sequence of PexA from 17 ICP1 isolates (Supplementary Table 5) from between 2001 and 2012. **b**, Praline alignment[2] of amino acid sequence conservation across Int from the characterized PLEs[3]. **c**, Cartoon depicting *attC* sites of characterized PLEs. PLE 1, PLE 3, PLE 4, and PLE 5 all integrate into the *V. cholerae* repeat[3] (VCR, grey triangles), of which there are over 100 sites within the super-integron[4] (red). PLE 2 integrates into a unique site in gene VCA0581[3] (yellow triangle) that shares no sequence similarity with the VCR.

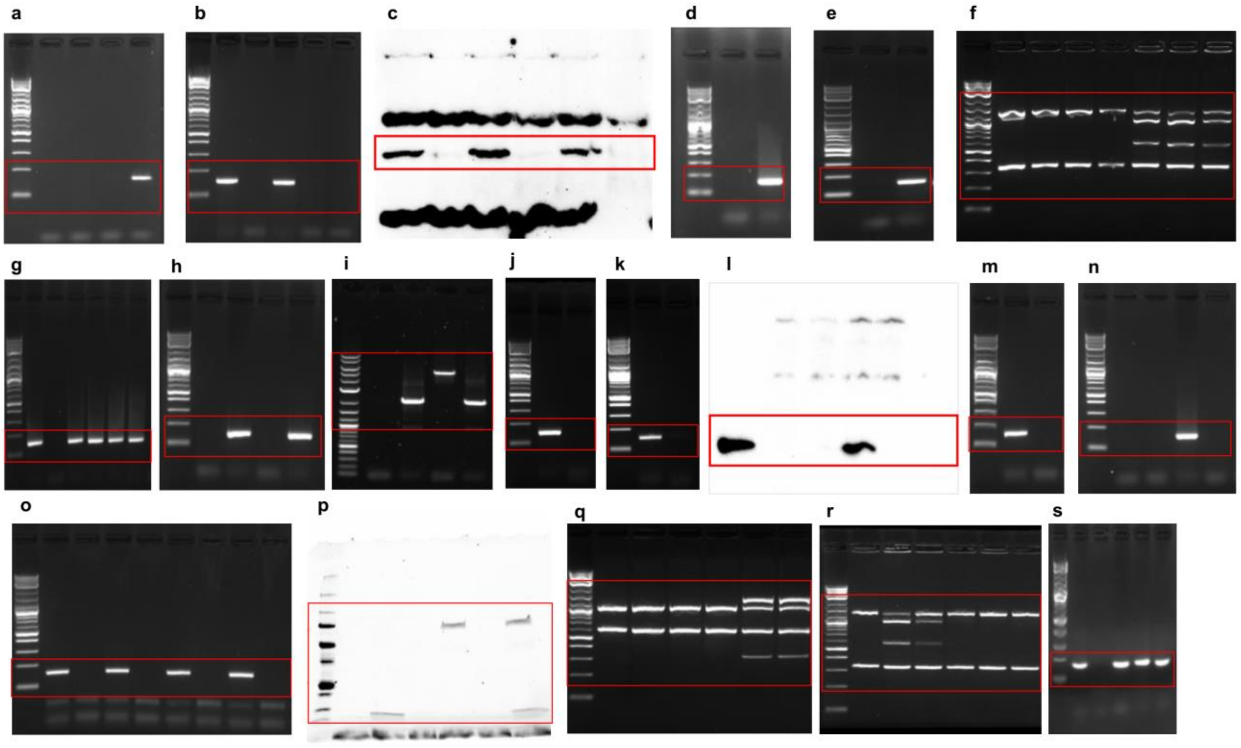

Supplementary Fig. 5 | Original gel images. a, Gel from Fig. 1c. **b**, Gel from Fig. 1d. **c**, Gel from Fig. 1e. **d**, Gel from Supplementary Fig. 1a. **e**, Gel from Supplementary Fig. 1b. **f**, Gel from Supplementary Fig. 1d. **g**, Gel from Fig 2a. **h**, Gel from Fig 2b. **i**, Gel from Supplementary Fig. 2. **j**, Gel from Fig. 3a. **k**, Gel from Fig 3b. **l**, Gel from Fig 3c. **m**, Gel from Supplementary Fig 3a. **n**, Gel from Supplementary Fig. 3b. **o**, Gel from Supplementary Fig. 3c. **p**, Gel from Fig. 4b. **q**, Gel from Fig. 4c. **r**, Gel from Fig. 4d. **s**, Gel from Fig. 5a.

Supplementary Table 1. Strains used in this study

Bacterial strains	Description	Source
**E7946**	*V. cholerae* O1, El Tor biotype; SmR, used as PLE(-)	Levine et al 1982[5]
**PLE 1**	E7946 containing PLE 1 integrated into VCR between VCA0329 and VCA0330	O'Hara et al., 2017[3]
**PLE 1 *Δint***	PLE 1 with an in-frame Spec-frt cassette replacing Orf1	This study
Δint* P_{tac}-*int	PLE 1 *Δint* with expression cassette modified from Dalia et al., 2014[6] for int under control of the P_{tac} promoter and a riboswitch integrated into the *V. cholerae lacZ* locus (*ΔlacZ*)	This study
Δint* P_{tac}-*EV	PLE 1 *Δint* with a P_{tac} promoter and a riboswitch integrated into the *V. cholerae lacZ* locus (*ΔlacZ*)	This study
**PLE 1 FLAG-Int**	PLE 1 with FLAG-tag fused to N-terminus of endogenous Int, Kanamycin resistance cassette inserted downstream of *int*	This study
**miniPLE**	E7946 containing PLE 1 *int* and Kanamycin resistance cassette integrated in VCR between VCA0329 and VCA0330	This study
**BTH1101**	*E. coli* adenylate cyclase knockout (*Δcya*), BACTH expression host	Lab collection
**E. coli BL21**	used for protein expression and purification	Lab collection
**PLE 1-Kan^R**	PLE 1, kanamycin resistance cassette inserted downstream of ORF 23	O'Hara et al., 2017[3]
**PLE 2-Kan^R**	E7946 containing PLE 2 interrupting VCA0581, Kanamycin resistance cassette inserted downstream of ORF 27	O'Hara et al., 2017[3]
**PLE 3-Kan^R**	E7946 containing PLE 3 integrated in VCR in between VCA0415 and VCA0416, Kanamycin resistance cassette inserted downstream of ORF 27	O'Hara et al., 2017[3]
**PLE 4-Kan^R**	E7946 containing PLE 4 integrated in VCR in between VCA0353 and VCA0354, Kanamycin resistance cassette inserted downstream of ORF 29	O'Hara et al., 2017[3]
**PLE 5-Kan^R**	E7946 containing PLE 3 integrated in VCR in between VCA0407 and VCA0408, Kanamycin resistance cassette inserted downstream of ORF 29	O'Hara et al., 2017[3]
**E7946 *ΔlacZ::att reporter***	Constitutively expressed *lacZ* cloned from *E. coli* flanked by part of a VCR that PLE 1 integrates into betweenVCA0462 and VCA0463 (*attC*) and the entire region between PLE 1 ORF23 and Int when PLE is circularized *(attP*)	This study
**PLE1 ΔORFs2-5**	PLE 1 ORF2- ORF5 replaced with an in-frame Spec-frt cassette	This study
**PLE1 ΔORFs7-14**	PLE 1 ORF7- ORF14 replaced with an in-frame Spec-frt cassette	This study
**PLE1 ΔORFs21-23**	PLE 1 ORF15- ORF20 replaced with an in-frame Spec-frt cassette	This study
**PLE1 ΔORFs15-20**	PLE 1 ORF21- ORF23 replaced with an in-frame Spec-frt cassette	This study
**KS441**	E7946 containing PLE 1 integrated into VCR between VCA0362 and VCA0363, used for *in vitro* recombination templates	O'Hara et al., 2017[3]

Supplementary Table 2. Primers used in this study

Primer	Sequence (5' - 3')	Application
**Zac14**	AGGGTTTGAGTGCGATTACG	qPCR FWD
**Zac15**	TGAGGTTTTACCACCTTTTGC	qPCR RV
**KS364**	CCGCTATCTTTCGAGGTAGC	circularization PCR FWD/in vitro *attP* template FWD/*attR* RV
**KS365**	GCTACTCTCCGTTAAATTCCG	circularization PCR RV
**ACM415**	CATGGCTTTGCGATGATGG	*in vitro attC* template FWD/*attR* FWD
**ACM416**	GGTGGTGCTTGGGATAACTC	*in vitro attC* template RV/*attL* RV
**KS289**	TTGGCGTTTACTAGATACTCGTC	*in vitro attP* template RV/*attL* template FWD
**KS587**	ATTCCGGGGATCCGTCGACC	amplify FRT-SpecR cassette
**KS586**	TGTAGGCTGGAGCTGCTTCG	amplify FRT-SpecR cassette
**ACM153**	CTGACATTGATTTCCCTCCG	PLE 1 Δint
**KS307**	GGTCGACGGATCCCCGGAATTGGCATATATATTCACACACGC	PLE 1 Δint
**KS308**	CGAAGCAGCTCCAGCCTACATAAGAAAAAGACCGCCCTATTG	PLE 1 Δint
**KS369**	CGTAACTAAATTGGTGGTGTGC	PLE 1 Δint
**KS372**	GGTGATTAATTGCTATACAAGTGG	PLE1 ΔORFs2-5
**KS371**	CGAAGCAGCTCCAGCCTACATAAAATAAACCGCCTCAATAGGG	PLE1 ΔORFs2-5
**KS477**	GGTCGACGGATCCCCGGAATACTACTCACTTTATATAGTTTTCCTTATGTTG	PLE1 ΔORFs2-5
**ks457**	CTGAAGATAATCTAACGATAGTTTATCTAACG	PLE1 ΔORFs2-5
**KS322**	AGCGGAGCTATTAAGTATGC	PLE1 ΔORFs7-14
**KS319**	GGTCGACGGATCCCCGGAATCATAAGGTTGGCTCCTCAATG	PLE1 ΔORFs7-14
**KS403**	CGAAGCAGCTCCAGCCTACAAATTGGCTCGACTTAATTTAA	PLE1 ΔORFs7-14
**KS379**	GGCTATATGTGCGTGTAATGC	PLE1 ΔORFs7-14
**KS334**	TACTCCCTTAGCAAGGTTGG	PLE1 ΔORFs15-20
**KS332**	GGTCGACGGATCCCCGGAATTGTTGGCATGTTCGTATTTCC	PLE1 ΔORFs15-20
**KS302**	CGAAGCAGCTCCAGCCTACATAAAGGCTAGAAAAATATGAACAAAG	PLE1 ΔORFs15-20
**KS304**	CCTGCGTTAACAGCTTCTGC	PLE1 ΔORFs15-20
**KS495**	GAAGATGGTGAGGCACTAGC	PLE1 ΔORFs21-23
**KS493**	GGTCGACGGATCCCCGGAATGATGTTCATATTTTCCTATCCTATCC	PLE1 ΔORFs21-23
**ACM363**	CGAAGCAGCTCCAGCCTACATAACTACTGGTTGCACAAGATTAACC	PLE1 ΔORFs21-23
**ACM26**	CTATGTGAACCAAAAGTTGAGCG	PLE1 ΔORFs21-23

Supplementary Table 2 (continued). Primers used in this study

Primer	Sequence (5' - 3')	Application
**KS501**	GGATCCCCAGCTTCGCGTCCGCGGTCTTTTTCTTAC GTGATTTTAAGC	miniPLE
**KS327**	CGGTGAACGCTCTCCTGAGTACAAGATTAACCATAT ATGAGCCG	miniPLE
**KS324**	GGACGCGAAGCTGGGGATCC	amplify KanR cassette
**KS325**	ACTCAGGAGAGCGTTCACCG	amplify KanR cassette
**ACM417**	GTTTGTCATCATCATCTTTATAATCCATATATATTCA CACACGCTTAATAAAAAG	PLE 1 FLAG-Int
**ACM418**	GATTATAAAGATGATGATGACAAACCAAAACTTTACA TATTTAGACGAGTATCTAG	PLE 1 FLAG-Int
**ACM321**	GGATCCCCAGCTTCGCGTCCTCGGTAGTATTAGGC TAACGCCCGCCTAAGGGGCTGGCAACGCATTAGCA CCAAACTCAAACACAACAACTGCAACGCGCAACGC AATTAATGTGAGTTAGCTCACTCATTAGGCACCCCA GGCTTTACACTTTATGCTTCCGGCTCGTATGTTGTG TGGAATTGTGAGCGGATAACAATTTCACACAGGATC CCGGGAGGAGGTAACGTAATGACCATGATTACGGA TTCACTGG	E7946 *ΔlacZ::att* reporter - gBlock containing *attC* and constitutive promoter
**KS853**	ATGACCATGATTACGGATTCAC	E7946 *ΔlacZ::att* reporter
**KS555**	TTATTTTTGACACCAGACCAACTGG	E7946 *ΔlacZ::att* reporter
**KS565**	CCAGTTGGTCTGGTGTCAAAAATAACTACTGGTTGC ACAAGATTAACC	E7946 *ΔlacZ::att* reporter
**ACM343**	GGTGTAGGCTGGAGCTGCTTCGGAGAGGCTTAACG CCTCTC	E7946 *ΔlacZ::att* reporter
**ACM344**	GAAGCAGCTCCAGCCTACACCACAATAAGCCAGAG AGCCTTAAG	E7946 *ΔlacZ::att* reporter
**ACM316**	CCTGCTAAGGAGGTAACAACAAGATGCCAAAACTTT ACATATTTAGACG	Δint P_{tac}-int
**ACM317**	CTCTCATCCGCCAAAACAGCTTACGTGATTTTAAGC TTGCGG	Δint P_{tac}-int
**ACM292**	TTTCGTCTTATGTGATCGATCGAGATATTGTGGTGA TG	ICP1 *ΔpexA*
**ACM293**	AAGACATCACCACAATATCTCGATCGATCACATAAG AC	ICP1 *ΔpexA*
**ACM301**	GCTATGACCATGATTACGCCACTAGACAATCTCACA AAAGAAG	ICP1 *ΔpexA*
**ACM289**	CGACGTTGTAAAACGACGGCCAAATAAGTTTGATGG CTGG	ICP1 *ΔpexA*
**ACM290**	GCAAGTTTTGGAGAATAATAATTTTAGAGGAAGGTG TGTATAATGAG	ICP1 *ΔpexA*
**ACM291**	GCAAGTTTTGGAGAATAATAATTTTAGAGGAAGGTG TGTATAATGAG	ICP1 *ΔpexA*

Supplementary Table 3. Plasmids used in this study

Plasmids	Description	Source
**P_{tac}-*pexA***	pMMB67E plasmid engineered to contain a riboswitch downstream of P_{tac} for inducible expression of PexA	This study
**P_{tac}-*EV***	pMMB67E plasmid engineered to contain a riboswitch downstream of P_{tac}, empty vector control	This study
**P_{bad}-*orf49***	pBAD derivative plasmid engineered to contain a riboswitch downstream of P_{bad} for inducible expression of ICP1 ORF49	This study
**P_{bad}-*orf50***	pBAD derivative plasmid engineered to contain a riboswitch downstream of P_{bad} for inducible expression of ICP1 ORF50	This study
**P_{bad}-*orf51***	pBAD derivative plasmid engineered to contain a riboswitch downstream of P_{bad} for inducible expression of ICP1 ORF51	This study
**P_{bad}-*orf52***	pBAD derivative plasmid engineered to contain a riboswitch downstream of P_{bad} for inducible expression of ICP1 ORF52	This study
**pE-SUMO-*int***	Vector to express 6xHisSumo-fusion protein, fused to N-terminus of Int	This study
**pE-SUMO-*pexA***	Vector to express 6xHisSumo-fusion protein, fused to N-terminus of PexA	This study
**T18-Int**	pUT18, T18 subunit of *cya* fused to C-terminus of *int*	This study
**T25-PexA**	pKNT25, T25 subunit of *cya* fused to C-terminus of *pexA*	This study
**T18-EV**	pUT18, T18 subunit of *cya*	Lab collection
**T25-EV**	pKNT25, T25 subunit of *cya*	Lab collection

Supplementary Table 4. Phage isolates used in this study

Phage isolates	Description	Source
**ICP1**	ICP1_2006_E *ΔCRISPR Δcas2-3*	This study
**ICP1 *ΔpexA***	ICP1_2006_E *ΔCRISPR Δcas2-3 ΔpexA*	This study
**ICP1 CRISPR+**	ICP1_2006_E	Seed et al., 2013[7]
**$ICP1_{2004}$**	ICP1_2004_A *Δcas2-3*	This study
**$ICP1_{2004}$ *Δorf49-52***	ICP1_2004_A *Δcas2-3 ΔORFs49-52*	This study
**$ICP1_{2004}$ ΔpexA**	ICP1_2004_A *Δcas2-3 ΔpexA*	This study
**$ICP1_{2005}$**	ICP1_2005_A *ΔCRISPR Δcas2-3*	This study
**$ICP1_{2005}$ *ΔpexA***	ICP1_2005_A *ΔCRISPR Δcas2-3 ΔpexA*	This study
**$ICP1_{2011}$**	ICP1_2011_A *ΔCRISPR Δcas2-3*	This study
**$ICP1_{2011}$ *ΔpexA***	ICP1_2011_A *ΔCRISPR Δcas2-3 ΔpexA*	This study

Supplementary Table 5. Phage isolates used in PexA sequence analysis

Phage Isolate	Year	Accession	Reference
**ICP1_2001_A**	2001	HQ641347	Seed et al., 2011[8]
**ICP1_2004_A**	2004	HQ641354	Seed et al., 2011[8]
**ICP1_2005_A**	2005	HQ641352	Seed et al., 2011[8]
**ICP1_2006_A**	2006	HQ641351	Seed et al., 2011[8]
**ICP1_2006_B**	2006	HQ641350	Seed et al., 2011[8]
**ICP1_2006_C**	2006	HQ641349	Seed et al., 2011[8]
**ICP1_2006_D**	2006	HQ641348	Seed et al., 2011[8]
**ICP1_2006_E**	2006	*	Seed et al., 2013[7]
**ICP1_2011_A**	2011	*	Seed et al., 2013[7]
**JSF01**	2001	KY883636	Naser et al., 2017[9]
**JSF02**	2001	KY883637	Naser et al., 2017[9]
**JSF04**	2001	KY065147	Naser et al., 2017[9]
**JSF05**	2002	KY883634	Naser et al., 2017[9]
**JSF06**	2002	KY883635	Naser et al., 2017[9]
**JSF13**	2009	KY883638	Naser et al., 2017[9]
**JSF14**	2011	KY883639	Naser et al., 2017[9]
**JSF17**	2012	KY883640	Naser et al., 2017[9]

*Sequenced genomes not available. *pexA* was amplified and sequenced using Sanger Sequencing.

1. Crooks, G., Hon, G., Chandonia, J. & Brenner, S. WebLogo: a sequence logo generator. *Genome Res* **14,** 1188–1190 (2004).

2. Bawono, P. & Heringa, J. PRALINE: A Versatile Multiple Sequence Alignment Toolkit. *Methods Mol Bio* **1079,** 245-262 (2014).

3. O'Hara, B. J., Barth, Z. K., McKitterick, A. C. & Seed, K. D. A highly specific phage defense system is a conserved feature of the *Vibrio cholerae* mobilome. *PLoS Genet.* **13,** e1006838 (2017).

4. Barker, A., Clark, C. A. & Manning, P. A. Identification of VCR, a repeated sequence associated with a locus encoding a hemagglutinin in *Vibrio cholerae* O1. *J. Bacteriol.* **176,** 5450–5458 (1994).

5. Levine, M. M. *et al.* The pathogenicity of nonenterotoxigenic *Vibrio cholerae* serogroup 01

biotype ei tor isolated from sewage water in brazil. *J. Infect. Dis.* **145,** 296–299 (1982).

6. Dalia, A. B., Lazinski, D. W. & Camilli, A. Identification of a Membrane-Bound Transcriptional Regulator That Links Chitin and Natural Competence in *Vibrio cholerae*. *mBiol.* **5,** e01028-31 (2014).

7. Seed, K. D., Lazinski, D. W., Calderwood, S. B. & Camilli, A. A bacteriophage encodes its own CRISPR/Cas adaptive response to evade host innate immunity. *Nature* **494,** 489–91 (2013).

8. Seed, K. D. *et al.* Evidence of a Dominant Lineage of *Vibrio cholerae*-Specific Lytic Bacteriophages Shed by Cholera Patiens over a 10-Year Period in Dhaka, Bangladesh. *MBio* **2,** e00334-10 (2011).

9. Naser, I. Bin, Hoque, M. M., Nahid, M. A., Rocky, M. K. & Faruque, S. M. Analysis of the CRISPR-Cas system in bacteriophages active on epidemic strains of *Vibrio cholerae* in Bangladesh. *Sci. Rep.* **7**, 14880 (2017). doi:10.1038/s41598-017-14839-2
